# Efficacy of Whole-Ventricular Radiotherapy in Patients Undergoing Maximal Tumor Resection for Glioblastomas Involving the Ventricle

**DOI:** 10.3389/fonc.2021.736482

**Published:** 2021-09-21

**Authors:** Kyung Hwan Kim, Jihwan Yoo, Nalee Kim, Ju Hyung Moon, Hwa Kyung Byun, Seok-Gu Kang, Jong Hee Chang, Hong In Yoon, Chang-Ok Suh

**Affiliations:** ^1^Department of Radiation Oncology, Yonsei Cancer Center, Yonsei University College of Medicine, Seoul, South Korea; ^2^Department of Neurosurgery, Yonsei University College of Medicine, Seoul, South Korea; ^3^Department of Radiation Oncology, Samsung Medical Center, Sungkyunkwan University School of Medicine, Seoul, South Korea; ^4^Department of Radiation Oncology, CHA Bundang Medical Center, CHA University, Seongnam, South Korea

**Keywords:** glioblastoma, ventricle, radiotherapy, temozolomide, MGMT

## Abstract

**Background and Purpose:**

Patients with glioblastoma (GBM) involving the ventricles are at high risk of ventricle opening during surgery and potential ventricular tumor spread. We evaluated the effectiveness of whole-ventricular radiotherapy (WVRT) in reducing intraventricular seeding in patients with GBM and identified patients who could benefit from this approach.

**Methods and Materials:**

We retrospectively reviewed the data of 382 patients with GBM who underwent surgical resection and temozolomide-based chemoradiotherapy. Propensity score matching was performed to compensate for imbalances in characteristics between patients who did [WVRT (+); n=59] and did not [WVRT (–); n=323] receive WVRT. Local, outfield, intraventricular, and leptomeningeal failure rates were compared.

**Results:**

All patients in the WVRT (+) group had tumor ventricular involvement and ventricle opening during surgery. In the matched cohort, the WVRT (+) group exhibited a significantly lower 2-year intraventricular failure rate than the WVRT (–) group (2.1% *vs*. 11.8%; P=0.045), with no difference in other outcomes. Recursive partitioning analysis stratified the patients in the WVRT (–) group at higher intraventricular failure risk (2-year survival, 14.2%) due to tumor ventricular involvement, MGMT unmethylation, and ventricle opening. WVRT reduced the intraventricular failure rate only in high-risk patients (0% *vs*. 14.2%; P=0.054) or those with MGMT-unmethylated GBM in the matched cohort (0% *vs*. 17.3%; P=0.036).

**Conclusions:**

WVRT reduced the intraventricular failure rate in patients with tumor ventricular involvement and ventricle opening during surgery. The MGMT-methylation status may further stratify patients who could benefit from WVRT. Further prospective evaluation of WVRT in GBM is warranted.

## Introduction

Glioblastoma (GBM) is the most common type of malignant primary brain tumor in adults and accounts for most deaths due to primary brain tumors (1). Currently, the standard treatment for GBM is maximal surgical resection followed by temozolomide (TMZ)-based concurrent chemoradiotherapy (2). Surgical management is the cornerstone of GBM treatment, and the goal is to minimize the residual postoperative contrast-enhancing volume, as this strongly correlates with survival (3–5). Various intraoperative mapping and monitoring techniques have emerged to increase the extent of resection while reducing the risk of new neurologic deficits (6–10). However, tumor location may limit the extent of surgery.

In newly diagnosed patients, GBM commonly involves the ventricular wall in 40–50% of cases (11–13). In such cases, surgeons are reluctant to surpass the ventricular wall due to a risk of iatrogenic tumor spread occurring through the ventricular system. Previous reports demonstrated the increased risk of leptomeningeal metastasis after surgical ventricular entry in high grade gliomas (14–16). Furthermore, patients with leptomeningeal and intraventricular tumor seeding in high grade gliomas are known to exhibit a dismal prognosis with a median survival of 2 to 6 months after being diagnosed with seeding metastases (14, 15, 17–19).

Despite the risk of seeding metastasis, our institution prioritized gross total tumor resection over ventricle wall preservation and have seen a relatively high median overall survival (OS) of 20–22 months (20–22), compared to the median OS of 15–18 months reported by other studies (23, 24). Considering the potential risk of intraventricular tumor seeding due to ventricular opening during resection, we electively treat the whole ventricle, with the ventricles opened, using intensity-modulated radiotherapy (IMRT). Whole-ventricular radiotherapy (WVRT) is commonly applied in cases of germ cell tumors (25) but is not commonly performed in GBM. Herein, we evaluated the efficacy and safety of WVRT in patients with GBM and to identify ideal candidates who may benefit from this treatment.

## Materials and Methods

### Patients

Between November 2005 and July 2019, the data of 433 consecutive patients with histologically confirmed GBM who were treated with TMZ-based chemoradiotherapy were retrospectively reviewed. Among these, patients with no post-treatment magnetic resonance imaging (MRI) data (n=9), initial leptomeningeal seeding (n=29), or gliomatosis cerebri who received whole brain radiotherapy (n=13) were excluded; thus, the data of 382 patients were analyzed. This retrospective study was approved by the Institutional Review Board (4-2020-1351).

### Tumor Location and Extent of Resection

All patients underwent preoperative MRI, including contrast-enhanced T1-weighted, T2-weighted, and T2-fluid attenuated inversion recovery sequences. Tumor ventricular involvement was defined as a contrast-enhancing lesion in contact with the ventricle.

The extent of resection and post-resection ventricle opening was evaluated using immediate postoperative MRI (performed within 48 h after tumor resection) and intraoperative findings during surgery. Gross total resection (GTR), subtotal resection (STR), and partial resection (PR) were defined as the absence of any contrast-enhancing lesions on immediate postoperative MRI, ≥90% of the tumor removed, and <90% of the tumor removed, respectively. Tumor specimens were examined for their DNA methylation status at the CpG islands on the MGMT promoter, as well as their IDH1R132H mutation status.

### Treatment

Patients underwent maximal tumor resection or stereotactic biopsy in cases not amenable to resection. Following surgical resection, concurrent TMZ-based chemoradiotherapy was administered to all patients within 4 weeks post-surgery. TMZ was applied at a dose of 75 mg/m2 every day during radiotherapy, followed by six cycles of adjuvant TMZ (150–200 mg/m2) for 5 days during each 4-week cycle (2, 24).

Radiotherapy was delivered as three-dimensional conformal radiotherapy (3D-CRT) until 2011; IMRT was widely adopted starting in 2012. The radiotherapy volume was defined according to Radiation Therapy Oncology Group guidelines, with some modifications (26). The gross tumor volume (GTV) was defined as the resection cavity and any residual contrast-enhancing tumor on immediate postoperative MRI with the addition of a 0.5–1-cm margin. The clinical target volume (CTV) included the peritumoral edema with a 1–1.5-cm margin. Based on the physician’s preference, the CTV was delineated by adding a 1.5-cm margin to the GTV regardless of the peritumoral edema in some cases. An additional 0.3-cm margin for setup uncertainty was added to the GTV and CTV to create the boost and initial planning target volume (PTV), respectively. For 3D-CRT plans, 46 Gy in 23 fractions was prescribed to the initial PTV and 14 Gy in 7 fractions was prescribed to the boost PTV. For IMRT plans, no additional PTV margin was added, and a simultaneous integrated boost technique was used to prescribe a total of 60 Gy and 51 Gy in 30 fractions to the GTV and CTV, respectively.

### Whole-Ventricle Radiotherapy

Since 2016, we have selectively applied WVRT in patients with exposed ventricles during tumor resection because of a concern for iatrogenic tumor spread through the ventricular system. All patients who received WVRT were treated with IMRT, and 45 Gy in 30 fractions was prescribed to the whole ventricle with the addition of a 0.3-cm margin. The whole ventricle was delineated per the ACNS1123 protocol (27).

### Follow-up and Failure Patterns

MRI was performed 4 weeks after the completion of CRT, every 12 weeks during adjuvant TMZ therapy, every 3 months for the first 2 years after the end of adjuvant TMZ therapy, and annually thereafter. Disease progression was determined based on radiologic, neurologic, and clinical findings, according to the Response Assessment in Neuro-Oncology criteria (28). All recurrences during the follow-up period were evaluated. The location of treatment failures was classified as local, outfield, or intraventricular failure, or leptomeningeal seeding. Local failure was defined when the epicenter of recurrence was within the initial PTV volume. Intraventricular failure was defined as recurrence within the ventricle and not including subventricular failures. Leptomeningeal seeding was defined as leptomeningeal involvement outside the ventricular system. Outfield failure was defined as recurrence outside the PTV volume but not intraventricular recurrence or leptomeningeal seeding. Radiation necrosis was confirmed if contrast-enhancing lesions with in the irradiated volume gradually decreased on more than two subsequent follow-up MRI studies performed and clinical symptoms improved. In contrast, contrast enhancing lesions that gradually increased on more than two subsequent follow-up MRI studies (with a size criterion of an increase of > 25% of the size of a measurable [> 1 cm] enhancing lesion according to the sum of the products of perpendicular dimensions) and deterioration of clinical symptoms were diagnosed as recurrences (29).

Peripheral blood counts were assessed every week during CRT and every 4 weeks during adjuvant TMZ therapy, and the change in blood cell counts during the course of treatment was analyzed.

### Statistical Analysis

Categorical variables were compared using the chi-square test or Fisher’s exact test, whereas continuous variables were compared using Student’s t-test or Mann–Whitney U test. Propensity score matching was performed to compensate for imbalances in the characteristics of patients who received and did not receive WVRT; we implemented a 1:2 nearest neighbor analysis, with a caliper width of 0.2 standard deviations of the logit distance measured using the R-package, “MatchIt.” The covariates used for matching included age, sex, Karnofsky performance score (KPS), MGMT methylation, ventricular involvement, ventricle opening, and resection extent.

Cumulative incidence estimates of each type of treatment failure, with death as a competing risk, were calculated and compared using Gray’s test. OS was defined from the date of surgery to the date of death. Progression-free survival (PFS) was defined from the date of surgery to the date of treatment failure or death. OS and PFS were estimated with the Kaplan–Meier method and compared using a log-rank test. Recursive partitioning analysis (RPA) was performed to stratify patients who did not receive WVRT according to their risk of intraventricular failure using the R-package, “rpart.” Variables such as age, sex, KPS, MGMT methylation, IDH1 mutation, ventricular involvement, ventricle opening, and extent of resection were included. Additionally, serial changes in blood cell counts and KPS were compared between groups using a linear-mixed model to account for missing values.

All statistical analyses were performed using SPSS version 25.0 (IBM SPSS Statistics, Armonk, NY), Graphpad Prism 8 (GraphPad Software, La Jolla, CA), and R software version 3.6.1 (R Foundation for Statistical Computing, Vienna, Austria). P-values <0.05 were considered statistically significant.

## Results

### Patient Characteristics

Among the 382 patients included, 59 received WVRT [WVRT (+) group] and 323 received localized radiotherapy [WVRT (–) group]. All patients in the WVRT (+) group had ventricular involvement with a contrast-enhancing lesion and ventricle opening during surgery (**Table 1**). GTR was performed in 66.5% of patients, and the extent of resection was similar in the two groups (**Table 1**). Although the IDH1 mutation rate was similar in the two groups, a significantly higher percentage of patients were not evaluated for IDH1 mutation status in the WVRT (–) group than in the WVRT (+) group. Significantly more tumors exhibited MGMT methylation in the WVRT (+) group than in the WVRT (–) group. All patients in the WVRT (+) group and 53.5% of patients in the WVRT (–) group received IMRT. Peritumoral edema was included in the radiotherapy field in most patients, but the rate was significantly lower in the WVRT (–) group than in the WVRT (+) group. The median dose delivered to the tumor bed was 60 Gy in 30 fractions and did not significantly differ between the two groups (**Table 1**). The median dose delivered to the whole ventricle was 45 Gy (range, 41.25–51 Gy) in 30 fractions.

**Table 1:** Patient characteristics in the pre- and post-matching cohorts.

Characteristics	Before matching				After matching		
	Total (n = 382)	WVRT (–) (n = 323)	WVRT (+) (n = 59)	*P* value	WVRT (–) (n = 111)	WVRT (+) (n = 59)	*P* value
Age, yrs, median (range)	58 (17-79)	58 (17-79)	58 (29-71)	0.574	58 (23-78)	58 (29-71)	0.82
Sex				0.025			0.399
Male	216 (56.5%)	191 (59.1%)	25 (42.4%)		56 (50.5%)	25 (42.4%)	
Female	166 (43.5%)	132 (40.9%)	34 (57.6%)		55 (49.5%)	34 (57.6%)	
KPS				0.459			0.419
<70	68 (17.8%)	60 (18.6%)	8 (13.6%)		22 (19.8%)	8 (13.6%)	
≥70	314 (82.2%)	263 (81.4%)	51 (86.4%)		89 (80.2%)	51 (86.4%)	
*MGMT* methylation				0.008			0.369
Unmethylated	236 (61.8%)	207 (64.1%)	29 (49.2%)		64 (57.7%)	29 (49.2%)	
Methylated	132 (34.6%)	102 (31.6%)	30 (50.8%)		47 (42.3%)	30 (50.8%)	
Not evaluated	14 (3.7%)	14 (4.3%)	0 (0.0%)		0 (0.0%)	0 (0.0%)	
*IDH1 *				1.000^*^			0.654^*^
Mutation	16 (4.2%)	14 (4.3%)	2 (3.4%)		7 (6.3%)	2 (3.4%)	
Wild type	273 (71.5%)	216 (66.9%)	57 (96.6%)		77 (69.4%)	57 (96.6%)	
Not evaluated	93 (24.3%)	93 (28.8%)	0 (0%)		27 (24.3%)	0 (0.0%)	
Ventricle involvement, T1-enhance				<0.001			NA
No	134 (35.1%)	134 (41.5%)	0 (0.0%)		0 (0.0%)	0 (0.0%)	
Yes	248 (64.9%)	189 (58.5%)	59 (100.0%)		111 (100.0%)	59 (100.0%)	
Ventricle involvement, T2-high				0.006			NA
No	37 (9.7%)	37 (11.5%)	0 (0.0%)		0 (0.0%)	0 (0.0%)	
Yes	345 (90.3%)	286 (88.5%)	59 (100.0%)		111 (100.0%)	59 (100.0%)	
Ventricle exposure during surgery				<0.001			NA
Not open	151 (39.5%)	151 (46.7%)	0 (0.0%)		0 (0.0%)	0 (0.0%)	
Opened	231 (60.5%)	172 (53.3%)	59 (100.0%)		111 (100.0%)	59 (100.0%)	
Extent of resection				0.283			0.968
Biopsy	19 (5.0%)	19 (5.9%)	0 (0.0%)		0 (0.0%)	0 (0.0%)	
Partial resection	28 (7.3%)	24 (7.4%)	4 (6.8%)		8 (7.2%)	4 (6.8%)	
Subtotal resection	81 (21.2%)	67 (20.7%)	14 (23.7%)		28 (25.2%)	14 (23.7%)	
Gross total resection	254 (66.5%)	213 (65.9%)	41 (69.5%)		75 (67.6%)	41 (69.5%)	
Radiotherapy modality				<0.001			<0.001
3DCRT	151 (39.5%)	151 (46.7%)	0 (0.0%)		48 (43.2%)	0 (0.0%)	
IMRT	231 (60.5%)	172 (53.3%)	59 (100.0%)		63 (56.8%)	59 (100.0%)	
Inclusion of peritumoral-edema				0.019			1.000
No	39 (10.2%)	38 (11.8%)	1 (1.7%)		2 (1.8%)	1 (1.7%)	
Yes	343 (89.8%)	285 (88.2%)	58 (98.3%)		109 (98.2%)	58 (98.3%)	
Dose, Gy, median (range)	60 (51.5-70)	60 (51.5-70)	60 (56-66)	0.216	60 (51.5-70)	60 (56-66)	0.836
Fraction, median (range)	30 (17-35)	30 (17-35)	30 (28-30)	0.588	30 (17-33)	30 (28-30)	0.219

3DCRT, 3-dimensional conformal radiotherapy; IMRT, intensity modulated radiotherapy; KPS, Karnofsky Performance Score; NA, not applicable; WVRT, whole ventricular radiotherapy.

^*^P value was estimated by comparing the percentage of IDH1 mutation vs. wild type or not evaluated.

To compensate for the imbalance in baseline characteristics between the WVRT (–) and WVRT (+) groups, propensity score matching was performed (**Table 1**). Because of the severe imbalance in the percentage of patients who had ventricular involvement and ventricles exposed during surgery, 111 patients were matched in the WVRT (–) group. Except for the radiotherapy modality, all variables were well-balanced following propensity score matching.

### Treatment Outcomes in the Matched Cohort

**Figure 1:**
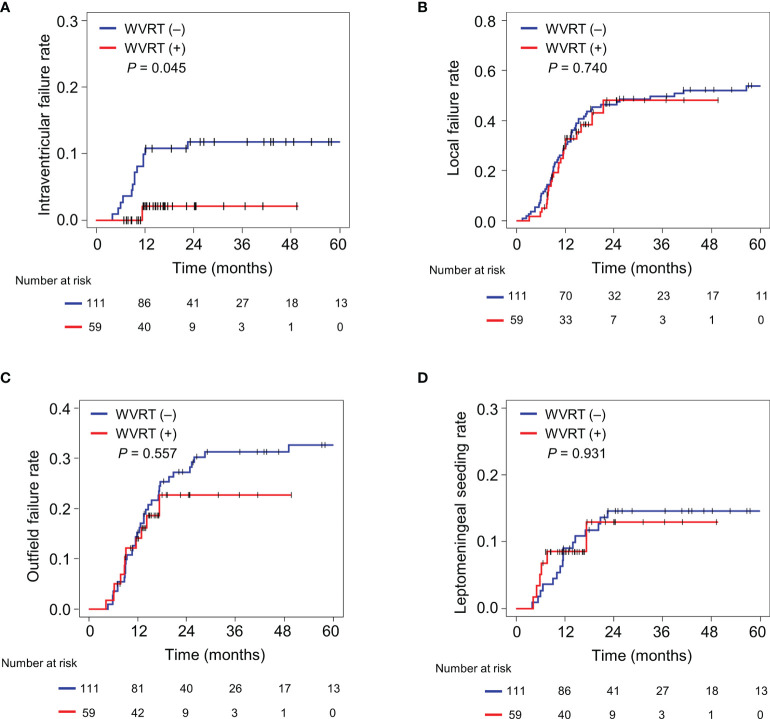
Cumulative incidence rates of intraventricular failure **(A)**, local failure **(B)**, outfield failure **(C)**, and leptomeningeal seeding **(D)** in the propensity score-matched cohort. The cumulative incidence rates were compared between the WVRT (–) group (n=111) and WVRT (+) group (n=59). WVRT, whole-ventricular radiotherapy.

The median follow-up time was 15.4 (range, 7.0–49.7) months in the WVRT (+) group and 47.6 (12.1–125.3) months in the WVRT (–) group. Intraventricular failures were noted in 22 patients, at a median of 9.5 (range, 3.2–112.8) months after diagnosis. One failure was noted in a patient in the WVRT (+) group and the remaining were noted in patients in the WVRT (–) group. The 2-year intraventricular failure rate was significantly lower in the WVRT (+) group than in the WVRT (–) group [2.1% (95% confidence interval (CI), 0.0–6.2%) *vs*. 11.8% (95% CI, 5.8–17.8%); P=0.045; **Figure 1A**]. However, there were no significant differences in the rates of 2-year local failure [48.1% (95% CI, 31.3–64.9%) *vs*. 46.4% (95% CI, 37.1–55.8%); P=0.740; **Figure 1B**], outfield failure [22.7% (95% CI, 12.8–40.2%) *vs*. 27.3% (95% CI, 19.0–35.7%); P=0.557; **Figure 1C**], and leptomeningeal seeding [12.9 (95% CI, 2.1–23.8%) *vs*. 14.6% (95% CI, 8.0–21.2); P=0.931; **Figure 1D**] between the WVRT (+) and WVRT (–) groups. Additionally, there were no significant differences in median PFS [15.4 (95% CI, 10.9–19.8) *vs*. 13.5 (95% CI, 11.4–15.6) months; P=0.577; **Supplementary Figure 1A**] and median OS [30 (95% CI, 17.6–42.4) *vs*. 19.6 (95% CI, 15.4–23.8) months; P=0.577; **Supplementary Figure 1B**] between the WVRT (+) and WVRT (–) groups.

### Risk Group Stratification of Intraventricular Seeding

**Figure 2:**
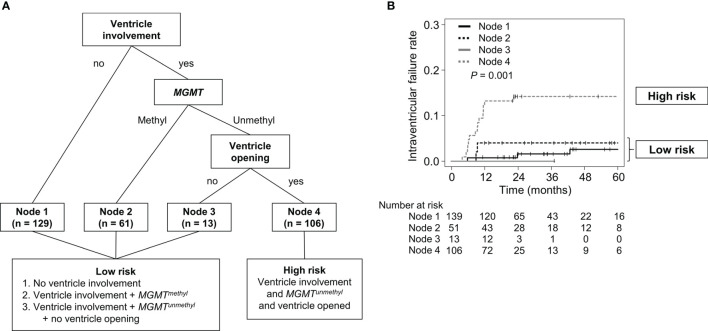
RPA classification according to the risk of intraventricular seeding. **(A)** Diagram of RPA classification in patients who did not receive WVRT. **(B)** Intraventricular failure rates of the RPA-classified nodes. RPA, recursive partitioning analysis; WVRT, whole-ventricular radiotherapy.

To further investigate the significant benefit of WVRT in terms of reducing the rate of intraventricular failure, we determined which subgroups were at higher risk of intraventricular failure and more likely to benefit from WVRT in an RPA. Only patients who did not receive WVRT were included (n=309) in the RPA. The patients were initially divided between those with and those without ventricular involvement of the contrast-enhancing lesion (**Figure 2A**). The MGMT methylation status was determined as the second most significant factor in dividing the patients. The final division was decided according to ventricle opening after surgery, which resulted in four terminal nodes (**Figure 2A**). The 2-year intraventricular failure rates for nodes 1, 2, 3, and 4 were 1.5% (95% CI, 0–3.6%), 3.9% (95% CI, 0–9.2%), 0%, and 14.2% (95% CI, 7.5–20.8%), respectively (P=0.001; **Figure 2B**). Nodes 1, 2, and 3, which had similar failure rates, were merged and classified as the “low-risk” group and node 4 was classified as the “high-risk” group (**Figures 2A, B**).

The high-risk group exhibited a significantly higher intraventricular failure rate than the low-risk group and showed higher rates of 2-year local failure [58.6% (95% CI, 49.2–68.0%) *vs*. 45.2% (95% CI, 38.3–52.2%); P=0.083; **Supplementary Figure 2A**], outfield failure [38.7% (95% CI, 29.4–48.0%) *vs*. 12.5% (95% CI, 7.9–17.1%); P<0.001; **Supplementary Figure 2B**], and leptomeningeal seeding [19.0% (95% CI, 11.5–26.5%) *vs*. 5.0% (95% CI, 2.0–8.1%); P=0.001; **Supplementary Figure 2C**]. Moreover, the high-risk group exhibited a significantly poorer median PFS [10.0 (95% CI, 9.0–11.8) *vs*. 15.7 (95% CI, 13.7–18.9) months; P<0.001; **Supplementary Figure 2D**] and median OS [16.3 (95% CI, 15.2–19.6) *vs*. 25.0 (95% CI, 22.4–29.8) months; P<0.001; **Supplementary Figure 2D**] than the low-risk group.

### Benefit of WVRT According to Risk Group Stratification and MGMT Methylation Status

**Figure 3:**
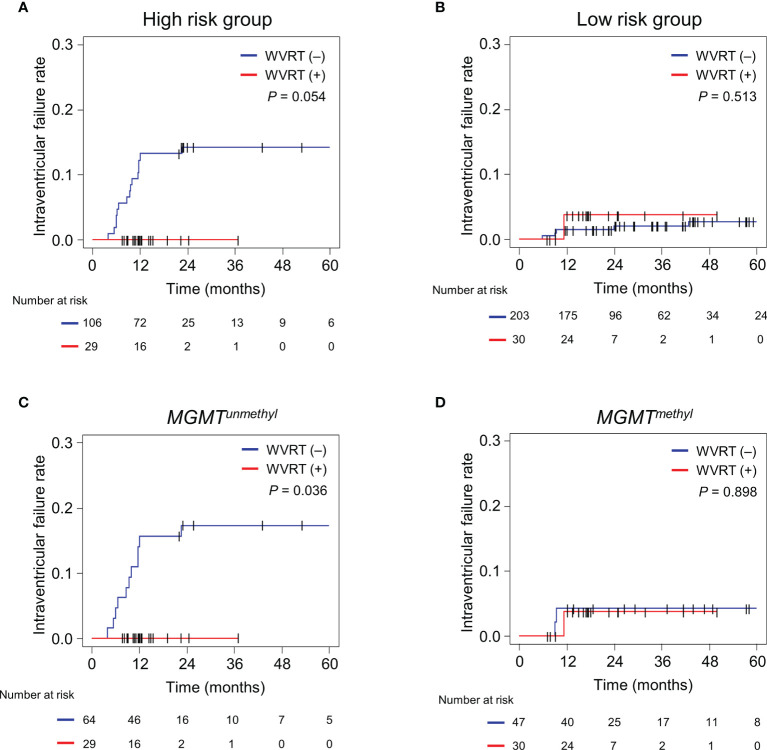
Cumulative incidence rates of intraventricular failure in different subgroups. **(A, B)** Intraventricular failure rates in the RPA-classified high-risk group (n=135; **A**) and low-risk group (n233; **B**). **(C, D)** Intraventricular failure rates in the propensity score-matched cohort (n=170) with MGMT-unmethylated GBM (n=93; **C**) and MGMT-methylated GBM (n=77; **D**). The cumulative incidence rates were compared between the WVRT (–) and WVRT (+) groups. RPA, recursive partitioning analysis; WVRT, whole-ventricular radiotherapy.

Next, the effect of WVRT on intraventricular failure was evaluated in each of the risk groups. The 2-year intraventricular failure rates for the WVRT (+) and WVRT (–) groups were 0% and 14.2% (95% CI, 7.5–20.8%; P=0.054; **Figure 3A**), respectively, in the high-risk group and 3.7% (95% CI, 0–10.9%) and 2.0% (95% CI, 0.1–4.0%; P=0.513; **Figure 3B**), respectively, in the low-risk group.

Considering that the MGMT methylation status was one of the main determinants for intraventricular failure, we further analyzed the effect of WVRT on the intraventricular failure rate in the propensity score-matched cohort (**Table 1**) according to the MGMT-methylation status. The 2-year intraventricular failure rates for the WVRT (+) and WVRT (–) groups were 0% and 17.3% (95% CI, 8.0–26.6%; P=0.036), respectively, in the MGMT-unmethylated subpopulation (**Figure 3C**) and 3.7% (95% CI, 0–10.9%) and 4.3% (95% CI, 0–10.0%; P=0.898), respectively, in the MGMT-methylated subpopulation (**Figure 3D**).

### Sites of Intraventricular Failure and Implications for WVRT Volume

**Figure 4:**
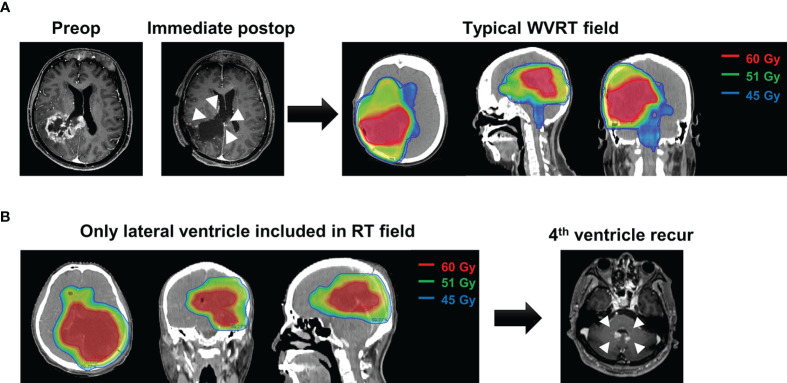
A representative case of intraventricular failure. **(A)** A typical case of WVRT after ventricle opening where the whole ventricle, including the lateral, third, and fourth ventricles are included. **(B)** A patient who did not receive WVRT but had the adjacent lateral ventricles included in the field. This patient experienced recurrence in the fourth ventricle 7 months post-surgery without local recurrence. WVRT, whole-ventricular radiotherapy.

To gain insight into the adequacy of the WVRT volume, we analyzed the sites of intraventricular failure. As mentioned above, 21 patients in the WVRT (–) group and one patient in the WVRT (+) group exhibited intraventricular failure. The common sites of failure included the lateral ventricle (7 of 22; 31.8%), fourth ventricle (7 of 22; 31.8%), and both the lateral and fourth ventricles (6 of 22; 27.3%; **Supplementary Table S1**). In five patients within the WVRT (–) group, the lateral ventricles, but not the fourth ventricle, were included in the radiotherapy field. Among these five patients, two experienced intraventricular failure and both of these had fourth ventricular involvement (**Figure 4**).

The failure patterns (**Supplementary Table S2**) showed that local failure was the predominant site of recurrence in both the WVRT (+) and WVRT (–) groups. Intraventricular failure was rarely observed as the sole site of failure (2/22, 11.1%); rather, it was more likely combined with local failure (7/22, 31.8%), leptomeningeal seeding (6/22, 27.3%), or both (7/22, 31.8%).

### Ventricle Opening During Surgery

Patients with ventricular involvement of a contrast-enhancing lesion had a significantly higher rate of ventricle opening during surgery than those without tumor ventricular involvement [87.0% *vs*. 11.9%; P<0.001]. Indeed, among patients with ventricular involvement [n=248] who received GTR, STR, and PR/biopsy, 95.5%, 86.0%, and 48.5%, had ventricle opening during surgery, respectively [P<0.001]. The rate of out-field recurrence was significantly increased in patients that had ventricle opening than in patients without ventricle opening [18.3% (95% CI, 13.1–23.5%) *vs*. 6.3% (95% CI, 0–14.6%); P = 0.032] but the rate of leptomeningeal seeding [9.5% (95% CI, 5.5–13.4%) *vs*. 3.1% (95% CI, 0–9.2%); P = 0.354] and intraventricuar seeding [7.6% (95% CI, 4.0–11.2%) *vs*. 0%; P = 0.199] did not significantly differ. Despite the significantly higher rate of ventricle opening in patients with a larger resection extent, the 2-year intraventricular failure rate of patients in the WVRT [–] group with tumor ventricular involvement [n=189] was similar among those who received GTR, STR, and PR/biopsy [8.6% (95% CI, 3.5–13.7%), 11.6% (95% CI, 2.0–21.2%), and 10.3% (95% CI, 0–21.4%), respectively; P=0.847; **Supplementary Figure 3A**]. Moreover, GTR was associated with a significantly higher median OS [21.5 (17.6–25.4) *vs*. 15.1 (95% CI, 13.7–16.5) *vs*. 15.7 (95% CI, 11.3–20.1) months; P<0.001; **Supplementary Figure 3B**] and median PFS [14.3 (95% CI, 10.9–17.7) *vs*. 9.1 (95% CI, 8.4–9.8) *vs*. 9.6 (95% CI, 7.5–11.7) months; P<0.001; **Supplementary Figure 3C**] than STR and PR/biopsy.

### Toxicity Following WVRT

**Figure 5:**
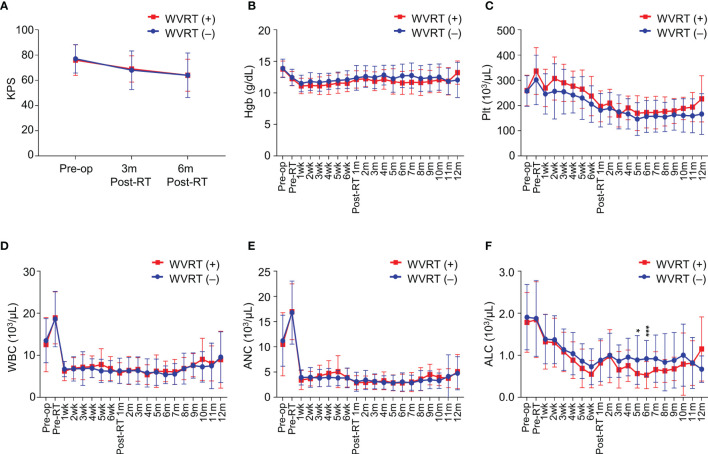
Performance status and blood cell counts during and after treatment in the propensity score-matched cohort (n=170). **(A)** Karnofsky performance score (KPS) before surgery, 3 months post-radiotherapy, and 6 months post-radiotherapy. **(B–F)** Serial values of blood test findings from before surgery to 12 months post-radiotherapy in terms of hemoglobin level **(B)**, platelet count **(C)**, white blood cell count **(D)**, absolute neutrophil count **(E)**, and absolute lymphocyte count **(F)**. ***P<0.001; *P<0.05.

Considering the increase in radiotherapy volume by including the whole ventricle, we compared the deterioration in performance, incidence of radiation necrosis, and changes in complete blood cell counts between the WVRT (+) and WVRT (–) groups. There was a significant decline in the KPS in both groups during the course of treatment (P<0.001), but there were no significant differences between the two groups in both the matched and whole cohorts (P=0.737; **Figure 5A** and **Supplementary Figure 4A**). The incidence of radiation necrosis did not significantly differ between the WBRT (+) and WVRT (–) groups in the matched cohort (8.5% *vs*. 7.2%; P = 0.769) and in the whole cohort (8.5% *vs*. 11.5%; P = 0.652). Among the 5 patients (8.5%) in the WVRT (+) group that experienced radiation necrosis, 4 patients were conservatively managed and 1 patient received bevacizumab for symptom control.

Next, we evaluated serial blood cell counts from baseline to 12 months post-radiotherapy. No significant differences between the WVRT (+) and WVRT (–) groups were observed in terms of hemoglobin level and platelet, white blood cell, and absolute neutrophil counts in both the matched and whole cohorts (**Figures 5B–E** and **Supplementary Figure 4B–E**). However, the absolute lymphocyte count (ALC) was significantly lower in the WVRT (+) group than in the WVRT (–) group at 5–6 months post-WVRT but not at other time points, and the ALC gradually recovered to a similar level as that in the WVRT (–) group (**Figure 5F**). In the whole cohort, the ALC was significantly lower during CRT and adjuvant TMZ therapy (P < 0.001), but it gradually recovered and did not significantly differ from that in the WVRT (–) group at 9 months post-radiotherapy (**Supplementary Figure 4F**).

## Discussion

To the best of our knowledge, this study provided the first data on the feasibility of WVRT for the treatment of patients with GBM who exhibit tumor ventricular involvement and ventricle opening during surgery. We found that WVRT can significantly reduce intraventricular seeding without increasing severe toxicity.

For GBMs involving the ventricle, ventricle opening is inevitable during maximal tumor resection. The concern for iatrogenic tumor spread due to ventricle opening during surgery discourages surgeons from performing maximal resection in GBMs involving the ventricle (14–16). However, the extent of tumor resection is an important prognostic factor in GBM (3, 4, 22, 30). Unlike tumor-specific prognostic factors such as the MGMT-methylation status, the extent of resection is a factor that can be controlled by surgeons. Therefore, surgeons should attempt maximal resection of the tumor. Additionally, surgeons should attempt to minimize the dissemination of tumor cells into the ventricle by opening the ventricle at the later stage of resection, after most of the main body mass has been removed, and closing the ventricular opening as soon as possible. In line with this policy, we perform maximal tumor resection even in tumors involving the ventricle, despite the risk of ventricle opening, and cover the whole ventricle in the radiotherapy volume.

The rate of intraventricular seeding was significantly lower in the WVRT (+) group than in the WVRT (–) of the propensity score-matched cohort. All patients in the matched cohort had ventricular involvement of the tumor and ventricle exposure during surgery, indicating the potential of WVRT to inhibit tumor spread after surgical ventricle entry. As WVRT significantly reduced intraventricular seeding, we further stratified patients to identify the subset of patients who may best benefit from WVRT. Tumor ventricular involvement was the strongest predictor of intraventricular seeding. Surprisingly, the MGMT-methylation status was the second most significant predictive factor for intraventricular seeding. Patients with MGMT methylation experienced a very low incidence of intraventricular seeding, despite ventricular involvement or ventricle opening. MGMT methylation is one of the most important prognostic and predictive markers in GBM (24, 30–32). Patterns of failure also differ between MGMT-methylated and MGMT-unmethylated GBMs, with MGMT-methylated tumors exhibiting higher distant failure rates than those seen in MGMT-unmethylated tumors (33, 34). This difference may be attributed to the higher sensitivity of MGMT-methylated tumors to TMZ/radiation and improved local control, which provides a higher chance of distant failure during follow-up. However, intraventricular seeding is more likely to be caused by direct tumor cell seeding during the surgical procedure. This is also supported by the early occurrence of most intraventricular failures during the first postoperative year. Among the seeded tumor cells in the ventricle, TMZ-sensitive MGMT-methylated tumor cells may be controlled through TMZ administration without WVRT, but TMZ-resistant MGMT-unmethylated tumor cells may have a better chance to repopulate the ventricle without WVRT, resulting in a higher rate of intraventricular failure.

Previous reports comparing treatment outcomes between tumors with and without ventricular involvement have demonstrated significantly poorer outcomes for tumors involving the ventricle (11, 13). In these studies, the rate of GTR in GBMs involving the ventricle was only half of that in tumors not involving the ventricle (11, 13). Along with the poor biological nature of tumors infiltrating the subventricular zone (SVZ) (35, 36), a less aggressive surgical approach may also contribute to the poor survival rates of patients with GBMs involving the ventricle. In this study, despite a higher rate of ventricular opening with a larger extent of surgery, patients who underwent GTR exhibited significantly higher PFS and OS than patients who underwent STR or PR/biopsy. This implies that maximal surgical resection should be attempted regardless of tumor ventricular involvement.

The dose applied for WVRT was 45 Gy in 30 fractions in most cases, which may seem to be insufficient to control GBM. However, we observed only one case of intraventricular failure among the 59 patients who received WVRT. Notably, this single case of intraventricular failure occurred after local failure. As tumor cells within the ventricle system seeded during surgery are less likely to be colonized, a relatively small dose may have been successful in eradicating the tumor cells, even in patients with MGMT-unmethylated GBMs. Interestingly, intraventricular seeding in the fourth ventricle was observed in some cases where only the lateral ventricles in proximity to the primary tumor were covered during radiotherapy (**Figure 4B**). Considering the high rates of recurrence in the lateral and fourth ventricles, the whole ventricular system should be covered when delivering WVRT.

Irradiating the whole ventricle was intended to target the cancer cells seeded within the ventricle by ventricular exposure during surgery. However, during WVRT, the SVZ, which has been suggested as a region where radioresistant cancer stem cells reside (35, 37), is also inevitably included in the radiation volume. Previous reports have demonstrated a benefit in PFS or OS with a higher radiation dose delivered to the SVZ (38–40). Chen et al. reported that the benefit of a higher SVZ dose was limited to patients who received GTR (39). However, there have also been contradictory results indicating that a higher SVZ dose does not correlate with improved survival after correcting for other clinicopathological factors (41). In this study, the reduction in intraventricular seeding with WVRT did not translate into a survival benefit. This may be attributed to the high proportion of local progressions among the treatment failures, where 80% of treatment failures were associated with local progression; WVRT could not reduce the rate of local failure. Moreover, the intermediate dose applied for WVRT may be insufficient to eradicate intrinsically radioresistant cancer stem cells in the SVZ. Lee et al. demonstrated that doses >59.4 Gy delivered to the SVZ were associated with improved PFS, unlike lower doses of 43 Gy (38). Considering these radioresistant cancer stem cells as a source of recurrence, better treatment strategies targeting this cell population may be needed to further improve treatment outcomes (42).

As including the whole ventricle, in addition to the tumor bed, increases the total radiotherapy volume, we examined the effect of WVRT on patient performance status and the change in blood cell counts following surgery. We did not observe any difference in patient performance status according to the receipt of WVRT in both the total and matched cohorts. Furthermore, the blood cell counts were similar between the two groups during follow-up, with the exception of the ALC, which was significantly lower in the WVRT (+) group than in the WVRT (–) group at 5–6 months post-radiotherapy in the matched cohort. However, the ALC gradually recovered in the WVRT (+) group and became similar to that in the WVRT (–). These findings of lower ALC can be explained by previous findings of a larger PTV volume significantly correlating with an increased incidence of acute severe lymphopenia in patients with GBM (43). Indeed, the increased irradiated volume in patients who receive WVRT can contribute to radiation-induced lymphopenia. Since acute severe lymphopenia has been demonstrated as a negative prognosticator for GBM and other solid tumors, efforts should be made to decrease the integral dose delivered to the brain (43, 44). Applying more advanced techniques in WVRT, such as proton therapy (45), may decrease the degree of lymphopenia and further improve the efficacy of WVRT in GBM.

Although this study provided data on a novel treatment approach for GBM, it has several limitations, which stem from its retrospective nature. The most significant weakness of this study was the shorter median follow-up time in the WVRT (+) group compared to the WVRT (–) group. Since WVRT was first applied after IMRT was introduced in the treatment of GBM, the WVRT (+) group comprised more recently treated patients, which led to a difference in the median follow-up time between the WVRT (+) and WVRT (–) groups. Although the median time to intraventricular seeding fell within the median follow-up time of the WVRT (+) group, the shorter follow-up time in the WVRT (+) group may have led to underestimation of the true incidence of intraventricular spread as failure may occur later on. Another limitation was the lack of data on patient neurocognitive function. The correlation between larger radiation volumes and poorer neurocognitive results have been demonstrated in previous studies (46, 47). However, neurocognitive test batteries were not routinely performed in the present study and the effect of WVRT on neurocognitive function were not able to be properly analyzed. Although no significant decline in KPS was observed in patients who received WVRT, language and verbal memory function may decline after WVRT due to the larger radiation volume.

In conclusion, WVRT significantly reduced the intraventricular failure rate in patients with GBMs involving the ventricle who had ventricles exposed during maximal tumor resection, especially in MGMT-unmethylated GBMs. Our data suggest that WVRT may be considered a new treatment option in these particular cases, and its efficacy should be prospectively evaluated in future studies.

## Data Availability Statement

The raw data supporting the conclusions of this article will be made available by the authors, without undue reservation.

## Ethics Statement

The studies involving human participants were reviewed and approved by Institutional Review Board of Severance Hospital (4-2020-1351). Written informed consent for participation was not required for this study in accordance with the national legislation and the institutional requirements.

## Author Contributions

KHK, JHC, HIY, and C-OS contributed to conception and design of the study. KHK and JY organized the database. KHK performed the statistical analysis. KHK wrote the first draft of the manuscript. NK, JHM, HKB, and S-GK wrote sections of the manuscript. All authors contributed to the article and approved the submitted version.

## Funding

This study was funded by the National Research Foundation of Korea (2020R1F1A1076287 to HIY).

## Conflict of Interest

The authors declare that the research was conducted in the absence of any commercial or financial relationships that could be construed as a potential conflict of interest.

## Publisher’s Note

All claims expressed in this article are solely those of the authors and do not necessarily represent those of their affiliated organizations, or those of the publisher, the editors and the reviewers. Any product that may be evaluated in this article, or claim that may be made by its manufacturer, is not guaranteed or endorsed by the publisher.
