# Supplementary material for: Efficacy of Whole-Ventricular Radiotherapy in Patients Undergoing Maximal Tumor Resection for Glioblastomas Involving the Ventricle
Source: Front Oncol. 2021 Sep 21;11:736482. doi: 10.3389/fonc.2021.736482 (PMC8490925; doi:10.3389/fonc.2021.736482)
Supplement: Supplementary file 1 [file DataSheet_1.docx]

Supplementary Material

Supplementary figures: 4

Supplementary tables: 2

# Supplementary Figures

**
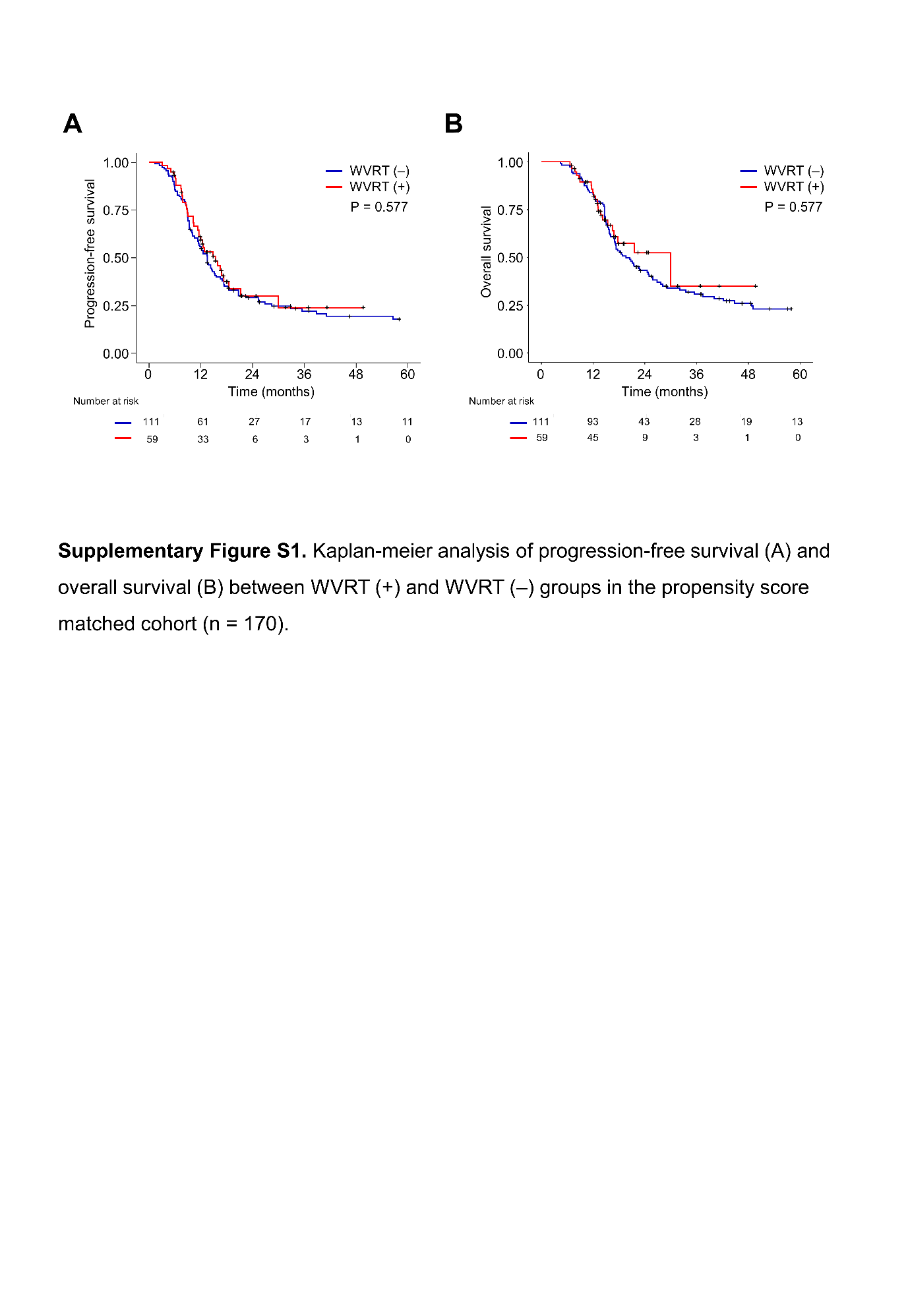
**

**Supplementary Figure 1.** Kaplan-Meier analysis of progression-free survival (A) and overall survival (B) between WVRT (+) and WVRT (–) groups in the propensity score matched cohort (n=170).

**
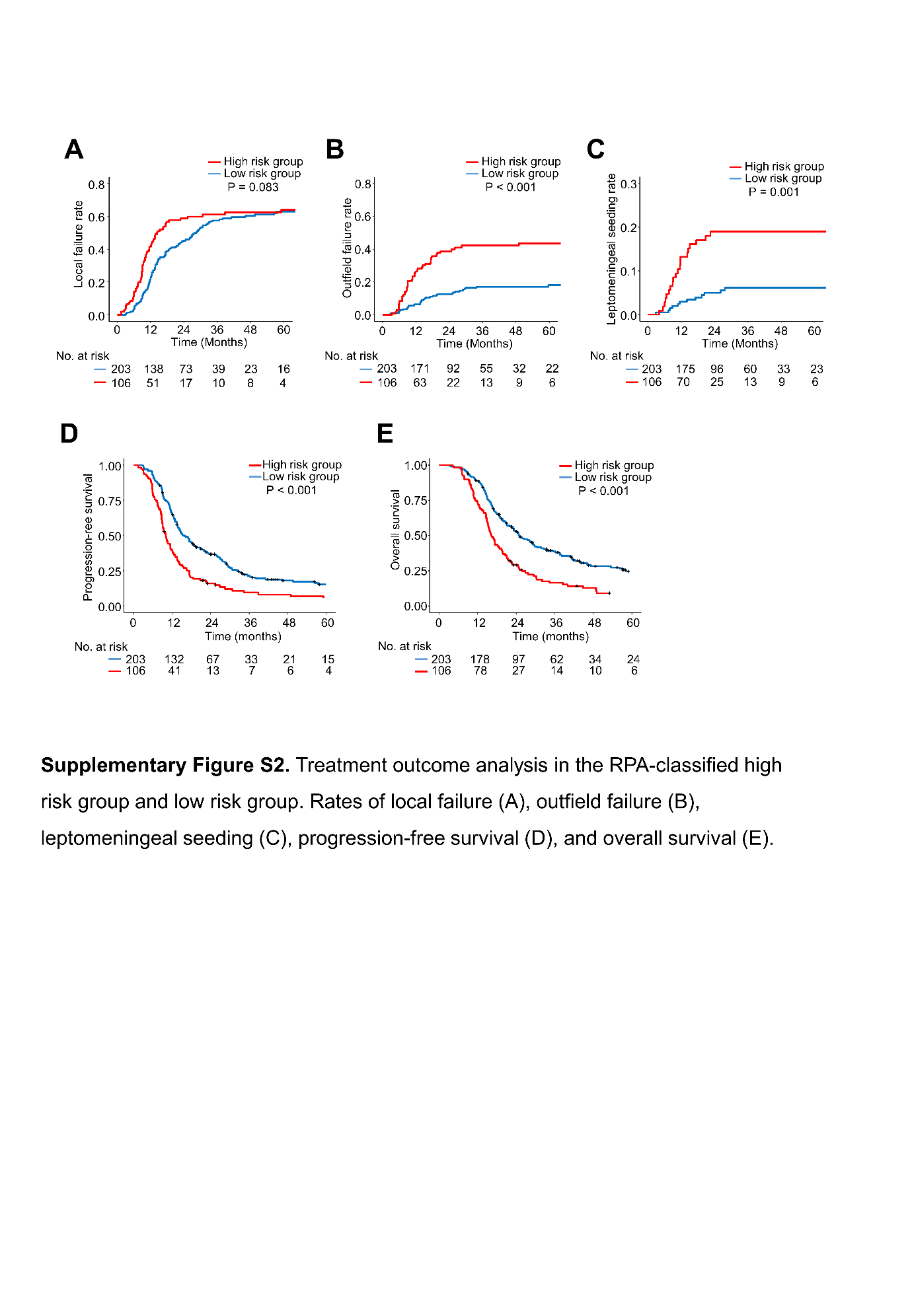
**

**Supplementary Figure 2.** Treatment outcome analysis in the RPA-classified high risk group and low risk group. Rates of local failure (A), outfield failure (B), leptomeningeal seeding (C), progression-free survival (D), and overall survival (E).

#
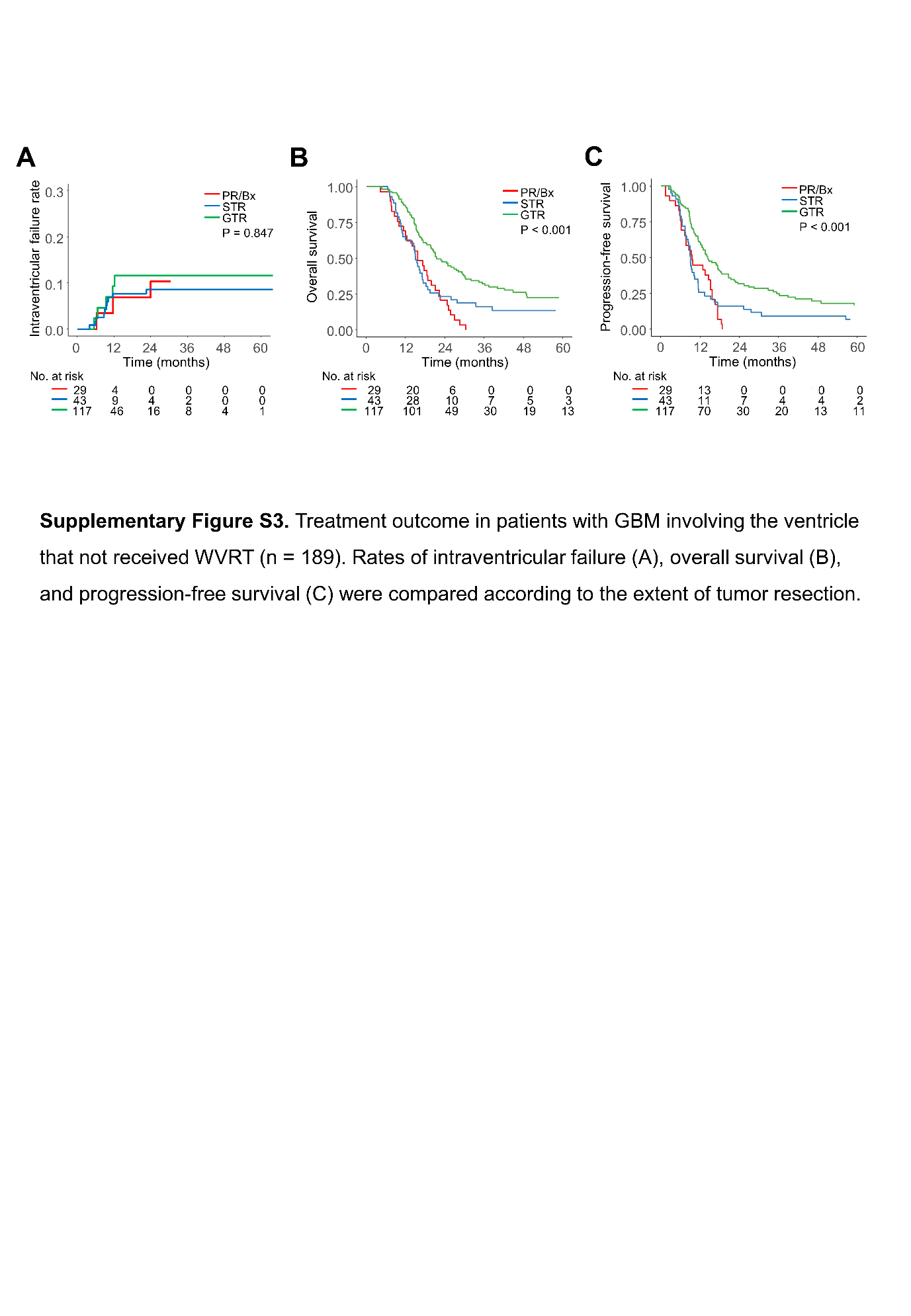


**Supplementary Figure 3.** Treatment outcome in patients with GBM involving the ventricle that not received WVRT (n=189). Rates of intraventricular failure (A), overall survival (B), and progression-free survival (C) were compared according to the extent of tumor resection.


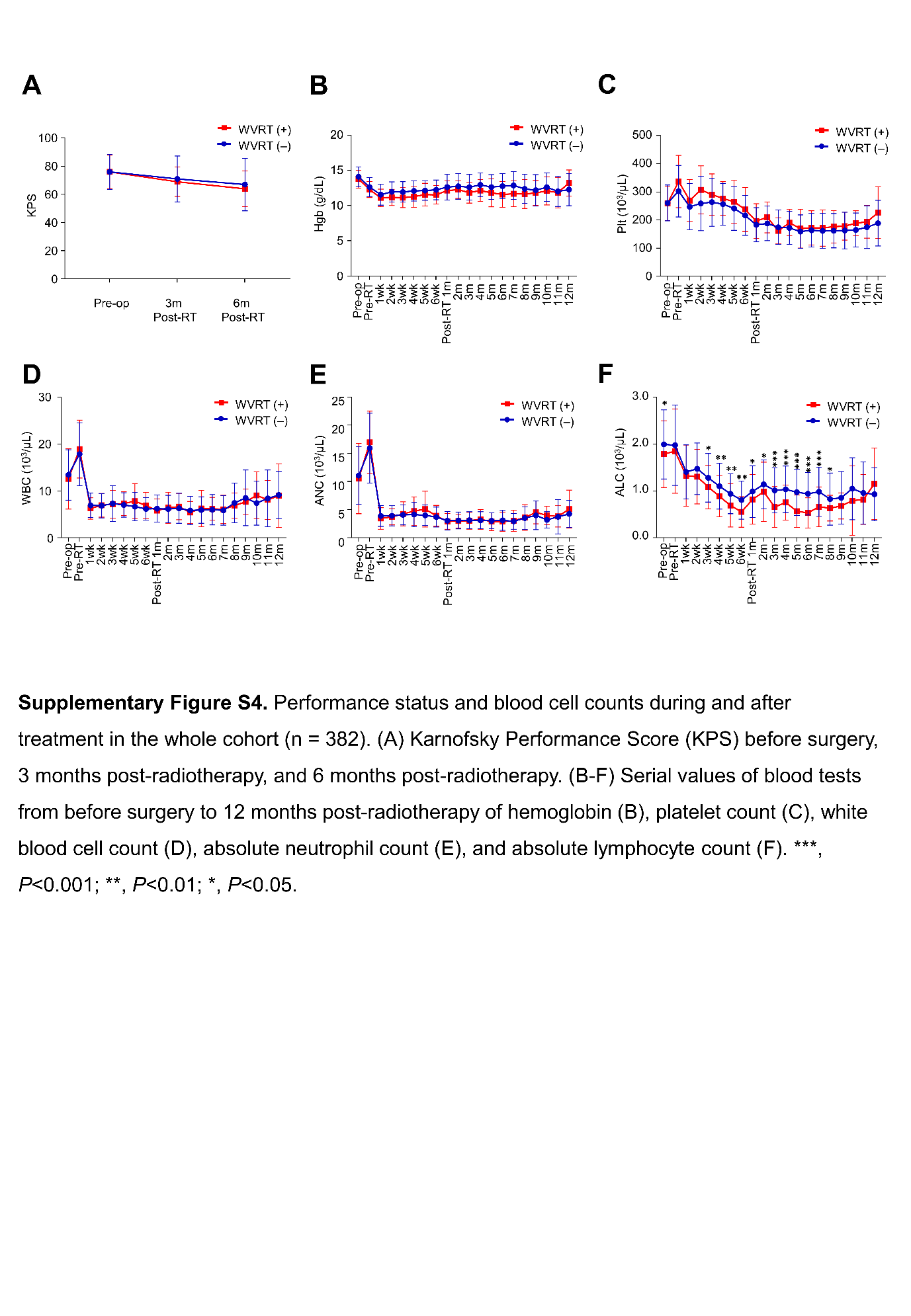


**Supplementary Figure 4.** Performance status and blood cell counts during and after treatment in the whole cohort (n=382). (A) Karnofsky Performance Score (KPS) before surgery, 3 months post-radiotherapy, and 6 months post-radiotherapy. (B-F) Serial values of blood tests from before surgery to 12 months post-radiotherapy of hemoglobin (B), platelet count (C), white blood cell count (D), absolute neutrophil count (E), and absolute lymphocyte count (F). ***, *P*<0.001; **, *P*<0.01; *, *P*<0.05.

# Supplementary Tables

**Supplementary Table 1. Sites of invtraventricular failure**

| **Intraventricular failure site** | **n (%)*** |
| --- | --- |
| Lateral ventricle only | 7 (31.8%) |
| 3^rd^ ventricle only | 1 (4.5%) |
| 4^th^ ventricle only | 7 (31.8%) |
| Lateral + 3^rd^ ventricle | 0 (0%) |
| Lateral + 4^th^ ventricle | 6 (27.3%) |
| Lateral + 3^rd^ + 4^th^ ventricle | 1 (4.5%) |

* Percentage among total intraventricular seeding (n=22).

One patient received WVRT and 21 did not receive WVRT.

**Supplementary Table 2. Patterns of failure in WVRT (+) and WVRT (–) groups**

|  |  | WVRT(+) n=59 |  |  |  | WVRT(–) n=323 |  |
| --- | --- | --- | --- | --- | --- | --- | --- |
|  | n | % of total | % of recurred patients |  | n | % of total | % of recurred patients |
| No recur | 30 | 50.8 |  |  | 82 | 25.4 |  |
| Recur | 29 | 49.2 | 100.0 |  | 241 | 74.6 | 100.0 |
| Local failure only | 13 | 22.0 | 44.8 |  | 154 | 47.7 | 63.9 |
| Outfield failure only | 3 | 5.1 | 10.3 |  | 26 | 8.0 | 10.8 |
| Intraventricular failure only | 0 | 0.0 | 0.0 |  | 2 | 0.6 | 0.8 |
| LMS only | 4 | 6.8 | 13.8 |  | 11 | 3.4 | 4.6 |
| Local failure + outfield failure | 7 | 11.9 | 24.1 |  | 17 | 5.3 | 7.1 |
| Local failure + LMS | 1 | 1.7 | 3.4 |  | 12 | 3.7 | 5.0 |
| Local failure + intraventricular failure | 0 | 0.0 | 0.0 |  | 7 | 2.2 | 2.9 |
| Outfield failure + LMS | 0 | 0.0 | 0.0 |  | 0 | 0.0 | 0.0 |
| Outfield failure + intraventricular failure | 0 | 0.0 | 0.0 |  | 0 | 0.0 | 0.0 |
| Intraventricular failure + LMS | 0 | 0.0 | 0.0 |  | 6 | 1.9 | 2.5 |
| Local failure + outfield failure+ intraventricular failure | 0 | 0.0 | 0.0 |  | 0 | 0.0 | 0.0 |
| Local failure + outfield failure + LMS | 0 | 0.0 | 0.0 |  | 0 | 0.0 | 0.0 |
| Local failure + intraventricular failure + LMS | 1 | 1.7 | 3.4 |  | 6 | 1.9 | 2.5 |
| Outfield failure + intraventricular failure +LMS | 0 | 0.0 | 0.0 |  | 0 | 0.0 | 0.0 |
| Local LMS + outfield failure + intraventricular failure + LMS | 0 | 0.0 | 0.0 |  | 0 | 0.0 | 0.0 |

Abbreviations, LMS = leptomeningeal seeding; WVRT = whole ventricular radiotherapy
